# Digitalization and Uncertainty in the University: Coherence and Collegiality Through a Metacurriculum

**DOI:** 10.1007/s42438-022-00324-1

**Published:** 2022-07-21

**Authors:** Mark William Johnson, Elena A. Suvorova, Alina A. Karelina

**Affiliations:** 1 University of Manchester, Manchester, United Kingdom; 2 Far Eastern Federal University, Vladivostok, Russia

**Keywords:** Metacurriculum, Transdisciplinarity, Uncertainty, Digitalization, Cybernetics

## Abstract

Recent initiatives to promote ‘digitalization’ in education exhorting increased digital literacy and ‘computational thinking’ have invited implementation research methods to transform curricula, teaching, and learning. While conceived as a movement from the present of education to an imagined future where envisaged curricula embrace data-oriented and computational practices across subjects, we ask: whose present? Whose future? We revisit the concept of a metacurriculum (first conceived in the 1990s) as a way of addressing this question, while not avoiding the challenges inherent in the adaptation of education to an increasingly complex postdigital environment. We argue that the principal challenge facing institutional and individual adaptation is increasing environmental uncertainty produced by technology, not deficiency in individual skills. Using the uncertainty concept, we present a practical co-designed and dialogical approach supporting the student and teacher journeys towards the transdisciplinary opportunities opened out by technology, based on a cybernetic model of intersubjectivity. We discuss the explanatory power of uncertainty in this context, focusing on the ways it can encourage dialogue, collegiality, and experimentation. Evidence for this is presented in a case study from a Russian University Business School where a large group of teachers co-designed and delivered a dialogical module on digitalization and interdisciplinarity over a period of 4 years—a collaboration ended by recent geopolitical events. In analyzing data from one of the central activities of this course, we focus on the teacher collegiality and the students’ mechanisms of selection in navigating the transdisciplinary space, and how these mechanisms may provide deeper insight into the dialogical underpinnings of education.

## Introduction: The Digitalization Metacurriculum

The concept of a ‘metacurriculum’ emerged in the 1990s in response to the rise of technology in education (Perkins et al. 1997; Lauer 1996; Koutselini 1997) referring to ways of supporting skills in ‘learning to learn’. In introducing her concept of ‘metacurriculum for critical thinking’, Lauer (1996) asked: ‘What can be done about the fragmentation and over-specialization of the traditional disciplines? How can students integrate what they learn for application to lifelong human issues?’

These questions seem more relevant and urgent now than they were in the 1990s. Technology appears to have contributed to an increase in specialization, while there is a simultaneous governmental concern about the need to address generic skills around technology across all disciplines. While a metacurriculum is an approach to address the transdisciplinary challenges, practical questions arise as to how to create the conditions where teachers can support it, the assessment system can recognize it, and students and their parents and teachers can be given the confidence that transdisciplinary approaches are worthwhile when specialization is increasingly fetishized.

Intellectual efforts to grapple with the nature of transdisciplinarity in its relation to knowledge sit alongside an awareness of deficiency in a traditional educational organization (Gibbs 2021; Barnett 2022; Green 2022). Barnett’s (2022) statement that ‘knowledge has retained a separateness even while it has understood itself as a machine through which humanity can exercise control over and an exploitation of the world’ suggests an intractable difficulty in navigating the space between conceptual categories, human understanding, and ecological flourishing.

We argue that this space is tractable through new perspectives on the relationship between technology and education and their effects on, and relationship with, their environment. Rather than focus on the categories of knowledge or technology, we instead focus on the *effects* of increasing specialization and technological proliferation on the environment. The digital world of the 1990s which Lauer’s questions addressed have, we suggest, been replaced with deeper postdigital questions concerning organic adaptative processes that react to environmental ambiguity and the manifest knottiness of digital logic which feeds back on political, social, educational, and biological concerns. The resulting environmental uncertainty often produces pathological defensiveness and institutional conservatism. However, it also creates the conditions for inquiry and dialogue.

We argue that these processes drive the digitalization agenda and feed the conservatism of the existing education system. Moreover, it seems that the world is becoming more uncertain and less predictable: not only by the increasing oscillation of weather dynamics, economic systems, and global disease but most recently the tragic geopolitical madness which ultimately has ended the collaboration that produced the current work. So our question can be framed: if we can understand the dynamics of uncertainty, is there a way of restructuring human relations around it so that the human dialogical relations that are central to learning can regulate rather than exacerbate uncertainty?

This requires:

1. An operationalizable theory of uncertainty and learning;
2. A pedagogical and organizational design for a metacurriculum;
3. A method for examining the dynamics of this design relating it back to theoretical principles.

To address 1, we draw on systems theory and cybernetics. Our approach to a metacurriculum partly echoes Lauer’s (1996) ideas about asking questions across the curriculum: teaching students to think in a transdisciplinary way, reflecting on their learning, while also introducing them to concepts around technology which present transdisciplinary connections. However, we differ from earlier approaches to a metacurriculum in considering that the educational challenge is now postdigital and uncertain. In place of a 1990s view of technology as instrumental to self-directed learning, we see technology as a fundamental contributing factor to the complexification of the environment to which learners, teachers, and institutions have to adapt. Since human development is intrinsically connected to the environment, we suggest that a metacurriculum is an acknowledgement that technology (digital or otherwise) is intrinsically connected to thinking and learning, much in the way that some philosophers of technology relate processes of ‘individuation’ to technology (Simondon 2016; Hui 2019).

In the context of this, a course was developed at the School of Economics and Management at the Far Eastern Federal University in Vladivostok, Russia. The school runs a range of Master’s programs with a yearly intake of about 250 students per year. In recognizing that these students left the university with little exposure to current and emerging science and technology, a project was created to design and run a transdisciplinary program which came to be called ‘Global Scientific Dialogue’.^1^

## Uncertainty and Postdigital Digitalization

The confidence in progress promoted by early digital pioneers (particularly in education) has been overshadowed by increasing uncertainty around technology in education and the manifest complexity of its social impact. The concept of the postdigital points to this relationship between technology and uncertainty, not least in the difficulty of finding a term which conveys the ‘messy and paradoxical condition of art and media after digital technology revolutions’ (Anderson et al. 2014). One way of characterizing this is to measure the proliferation of technological options, which must (as with all proliferation) lead to increasing difficulty in choosing and coordinating effective action. Whether technology is seen as an ‘extension’ of existing capabilities (McLuhan 2012), amplifications (Illich 2001), reordering of nature (Heidegger 1978), or changes to the means of production and social relations (Marx 1990), an additional option means that the complexity of choosing whether to use a new method or stick with the old method increases.

Leydesdorff (2021) has recently pointed to the relationship between this increase in technological options, and the increase in ‘maximum entropy’ in the social system, citing evidence from ecology (Brooks and Wiley 1988). In the face of the explosion of technology in the environment produced by the Internet, AI, and social media, the challenge for individuals is to ask: which options do I choose to organize myself effectively? Equally, universities must ask: which options do we choose to maintain coherence and viability within our institution? The institutional response, fearing the chaos of personal technological choice, can result in the removal of technological options from teachers and students. While this makes the institutional technological environment manageable, the risk is that institutional decisions (for example, prohibiting the use of certain tools) weigh on educational practices typified by exasperated responses by individuals such as: why can’t we just use Google docs?

In the wake of this technologically induced uncertainty, universities, governments, and schools have been promoting ‘digitalization’ on the curriculum, alongside skills such as ‘computational thinking’ (Wing 2008). However, efforts to instill this or even to define it have proved challenging (Lye and Koh 2014; Weintrop et al. 2016; Shute et al. 2017). The focus of such efforts often involves teaching technical skills within the established frameworks of the traditional curriculum. Our uncertainty perspective looks beyond this, and to the challenges that face any teacher or learner in the face of increasing technological options and environmental complexity. In the push for teaching new skills, the space for dialogue to negotiate this uncertainty is crowded out by a continual exhortation of ‘future skills’, yet without recognition that all teachers and learners face similar dilemmas in navigating complexity. Exhortation or imposition of new requirements is likely to exacerbate the problem.

**Fig. 1:**
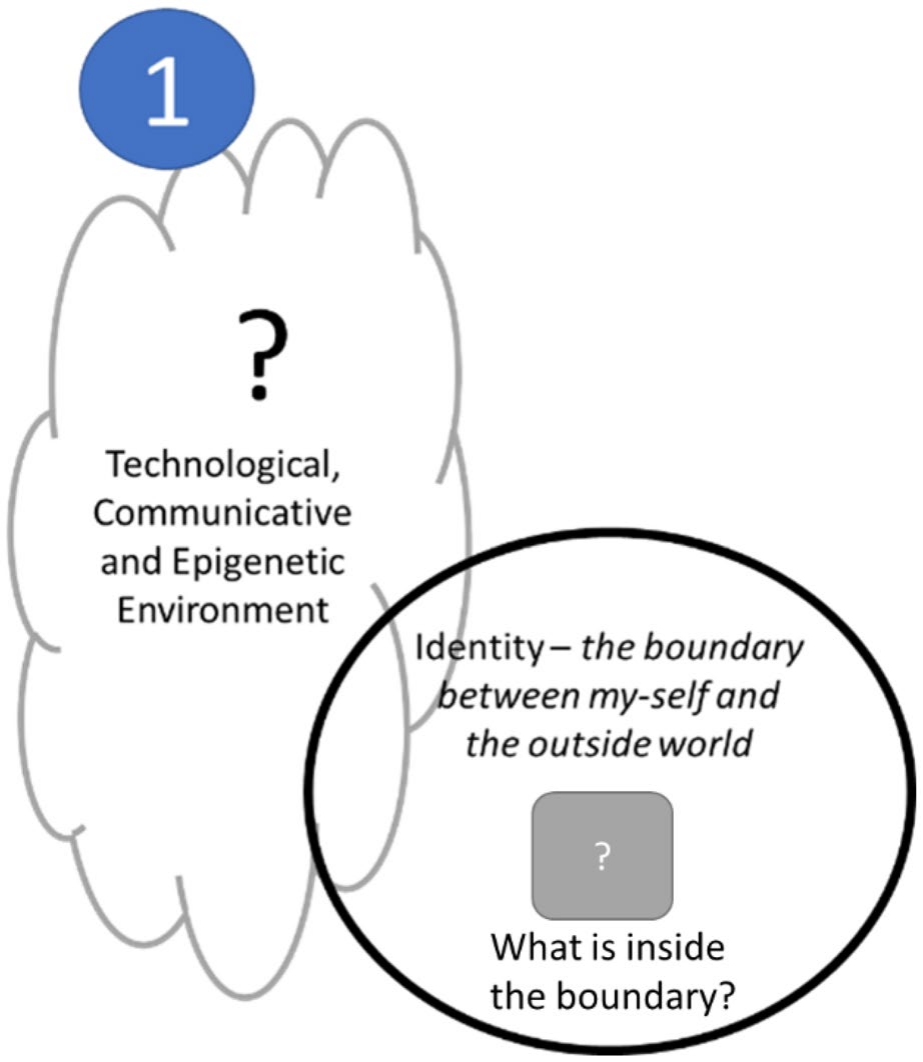
The uncertainty of a boundary in an environment

To explain our position, we draw on a simple cybernetic example. Consider that any viable entity—an organism, a person, or an institution—must maintain a distinction between themselves (their identity) and their environment (c.f. Beer 1994a; Spencer-Brown 2010). Such a distinction, shown in Fig. 1, must contain uncertainty: following Gödel’s First Incompleteness Theorem (1964), what belongs within the boundary and what belongs outside are unprovable within the boundary. As both Beer’s (1994b, 2007) management cybernetics and Luhmann’s (1996) social systems theory point out, there is inherent ambiguity in any boundary: between what is ‘marked’ and what is ‘unmarked’ (Spencer-Brown 2010).

**Fig. 2:**
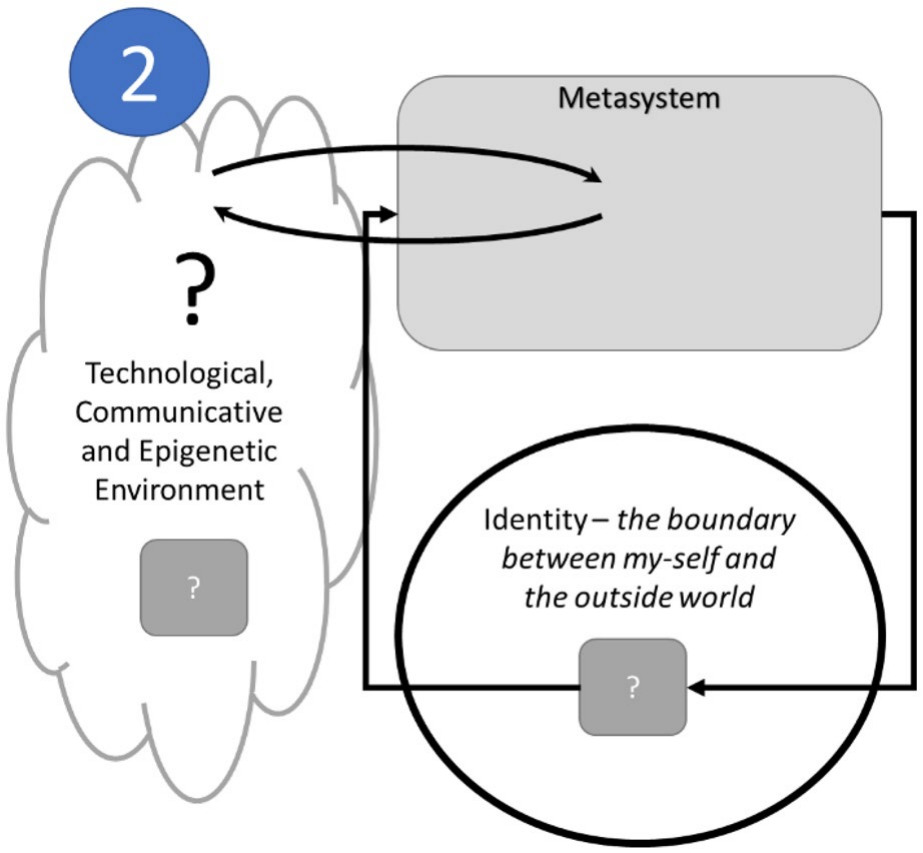
Balancing internal and external uncertainty with a metasystem

Uncertainty results from unmanaged complexity, and Beer (1994a) argues that since organisms and people are viable and adaptive, there must be a ‘meta-system’ that connects to the environment and balances the uncertainty within the system with the uncertainty that pertains to the environment. This elaboration of Fig. 1 is shown in Fig. 2.

A further elaboration of Fig. 2 can be produced. An educational model can be constructed from this principle where individuals (teachers and learners) are seen as viable, adaptive systems surviving in an uncertain or ambiguous environment. Adaptation to the environment means balancing and negotiating the uncertainties in the environment with the uncertainties within the person, which we conceive as ‘internal’ uncertainty, and ‘external’ uncertainty, and which sociologists such as Luhmann and Parsons have called ‘double-contingency’ (Luhmann 1996). Creative activities and conversation allow for inner uncertainties to be expressed so that a dialogic space (Wegerif 2010) can be opened to allow students to deepen their inquiries, both within a course and beyond it.

**Fig. 3:**
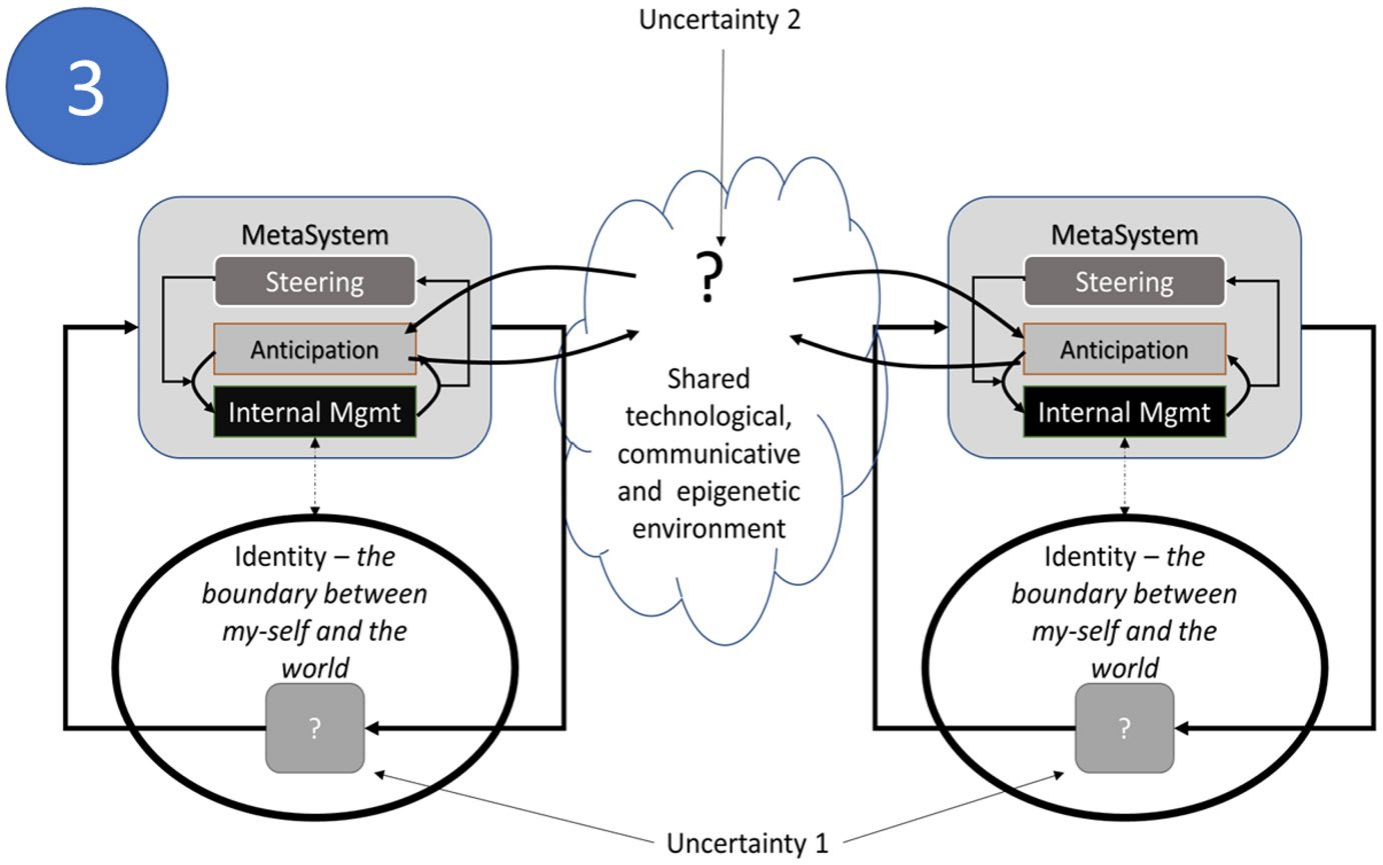
Uncertainty management in intersubjective relations

In Fig. 3, two people structured in this way are in dialogue. Here, the metasystem is broken down into three components: a component to manage internal uncertainty (labelled ‘Internal Mgmt’), a component to engage in, model, and anticipate the environment (‘Anticipation’), and a component that balances the relationship between the other two (‘Steering’). In this process, the anticipation component attempts to build predictive models of, and make interventions, in the environment, identifying threats and opportunities to which the individual must internally adapt.

Such a model forms the basis for our asking: what is a metacurriculum? What is digitalization? What is dialogue? While accepting that all models are wrong, as a compass for taking educational action, modelling allows us to design a set of interventions and to interpret their results. We have found it particularly valuable in thinking about digitalization because it situates technology within the dynamic balance between external uncertainty and internal uncertainty, rather than as a mind-independent entity. This approach also reflects the insights into the physiological organization of Beer’s (1994a) management cybernetics, while more recently, Noble (2002, 2008) has argued that such processes characterize what he calls ‘middle-out’ development in cellular systems (as opposed to top-down or bottom-up). Central to the middle-out dynamic is communication—whether between cells, or the dialogue between individuals in an organization.

As a middle-out process, digital phenomena exist in the environment, and result in increasing uncertainty (uncertainty 2) to which individuals and institutions have to adapt through dialogue. Learning occurs within this dialogical adaptation—not focused on technology itself, but on the uncertainty it generates. Unlike other approaches to digitalization, technology is not there as a ‘thing to be taught,’ but a catalyst in the environment to coordinate expectations and discussion around inherent uncertainty. Used in this way, digitalization can be exploited as a way of realizing a deeper meaningful coordination of expectations between teachers and students.

Since each individual—teacher or student—has to balance their internal uncertainty (uncertainty 1) with the uncertainty of the environment, this environmental intervention can be seen to cause a disruption to each student’s internal uncertainty management. In the context of an invitation to discuss, experiment, and create, those aspects of internal uncertainty which had been managed by the individual’s metasystem are projected into the social domain through activities and presentations. Teachers can facilitate this process, but there are many issues which arise where they too can be uncertain. Through this exploitation of uncertainty, combined with creative activities, the loop between thought, communication, and action can be explicitly operationalized through institutional structures and pedagogy.

In this way, the different environmental contexts for dialogue (for example, topics, activities, technologies, face-to-face or online) must be considered, since each context carries different levels of uncertainty: understanding the institutional dimensions of uncertainty sheds further light on the ways learners and teachers navigate and construct a dialogic space. In the conventional educational system, the principal environmental context is provided by the curriculum.

## The Institutional Dimensions of Uncertainty

Viewed from the perspective of uncertainty, the curriculum provides a central regulating function within the organizational structures of educational institutions. It attenuates the complexity of the world, dictating that, for example, in the Maths class, one does not usually perform star jumps. Curriculum is the means whereby conversations in the institution are coordinated, courses designed, staff hired, and students recruited and assessed.

While appreciating the necessity for curriculum attenuations, an educational approach more closely oriented to a metacurriculum might create the opportunity for physical movement in the maths class (perhaps, for example, by studying AI processing of movement patterns). This can only happen by loosening the structural constraints of the institution. In this spirit of developing innovation and educational experiment within the Russian management school, we were granted:

- Freedom to change the structure of teaching, with 20 teachers acting as facilitators coordinating small groups of students in activities shared across the whole cohort;
- Flexibility in assessment which was portfolio-based and assessed using a ‘patchwork text’ (Winter 2003) approach;
- Flexibility in the timetable where the course ran in a concentrated block of 8 full days, rather than spread over 14 weeks;
- Flexibility in the use of the campus where (before going online in 2020) students were divided into small groups in 20 classrooms operating simultaneously.

As Beer (1994b, 2007) points out, all institutions are like physiological bodies, having ‘functionally differentiated’ components and that continually coordinate their operations with one another. While these functions serve to manage different areas of uncertainty, they also serve to reinforce the ‘reality’ of the distinction between the different practices and artifacts of education: classrooms, timetables, curricula, textbooks, computers, teachers, budgets, buildings, catering, etc. This process of reinforcement is particularly noticeable within curriculum subjects, and it presents one of the challenges for any viable metacurriculum.

**Fig. 4:**
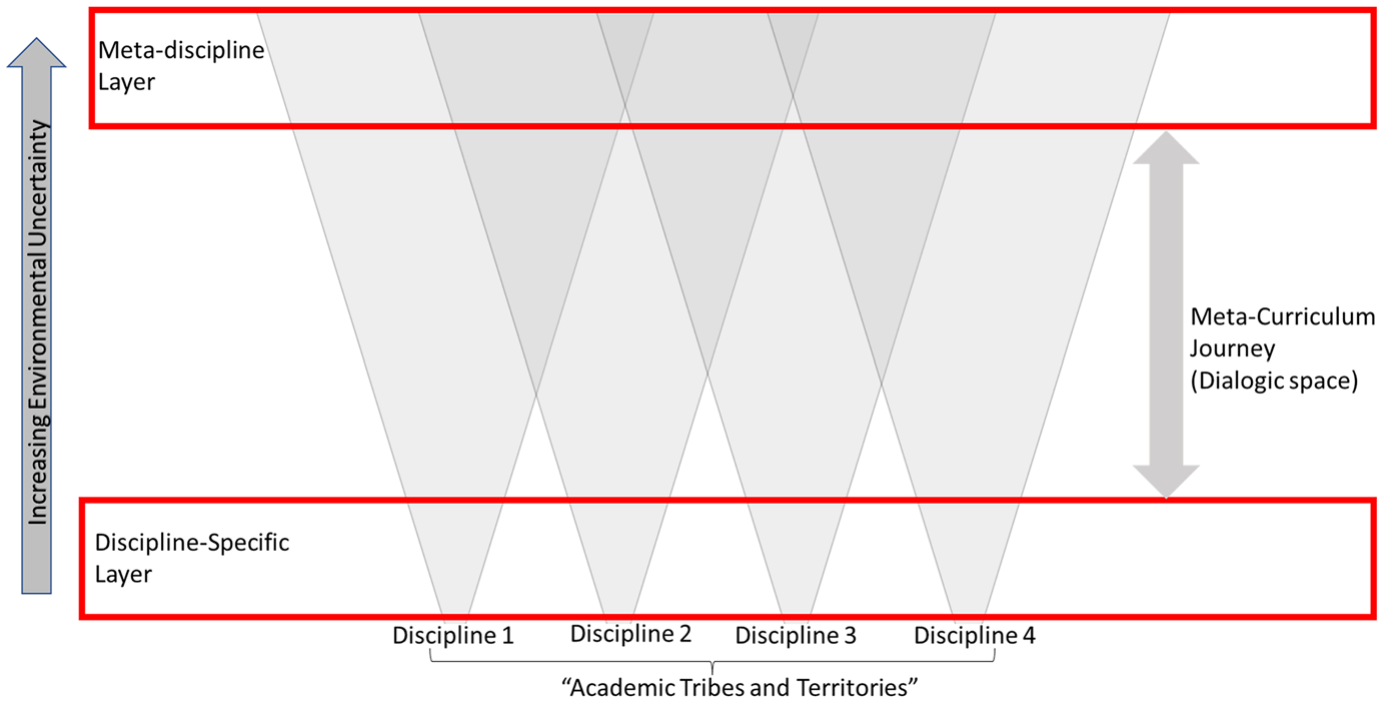
Technology, disciplinary specifics, and transdisciplinarity

This can be illustrated in Fig. 4. This shows the varying specifics and generalities that relate institutionally organized disciplines to technologies and transdisciplinarity. Traditional disciplinary identity is maintained at the bottom of the diagram with specific communities and discourses. As Trowler and Becher (2001) have described, disciplinary distinctions are maintained with other disciplines as ‘academic tribes’. The ‘cones’ from each discipline from bottom to top represent increasing technological options, complexity, and environmental uncertainty. As options increase, so the cross-over of techniques, practices, and discourse increases, as illustrated by the overlapping of cones.

The top level of Fig. 4 represents the kind of generalized transdisciplinary level which is sometimes advocated by proponents of digitalization as a future where disciplinary distinctions are dissolved into technical practice. However, while such a transdisciplinary level arguably exists, Fig. 4 attempts to show that this transdisciplinary level is part of an overall dynamic that connects specifics of knowledge production to generalities. It is the journey of students and teachers from the specifics of disciplines to transdisciplinarity which counts, and it is in supporting this journey that the challenge of digitalization, and a metacurriculum to support it, rests.

Within specific discourses (at the bottom of the diagram), uncertainties can be kept at a minimum, reinforced by traditional methods of teaching, assessment, timetable, and the need to concentrate on disciplinary specifics. While technologies may be used in specific ways in disciplines, there is often little overlap in tool use between Anthropology and Chemistry, for example.

The movement beyond the uncertainty management of particular groups requires the establishment of new kinds of intersubjective engagement, free from the constraints which operate at the lowest level of curriculum-specific organization. This in turn increases the space for potential dialogue about a broader range of topics. For example, to talk about the specifics of technology or data analysis is distinct from talking about interdisciplinarity or creativity. In each case, there are different levels of uncertainty which cross over other disciplines.

Examining the dynamics across this range between disciplinary specifics and generalities has been the focus of our method with an emphasis on dynamics in intersubjective engagement over content delivery. This necessitates not only the exploitation of uncertainty to build trust between students, but also to establish collegial relations and practice change among staff.

## Collegiality, Uncertainty, and Linearity: Whose Present? Whose Future?

Within an uncertain context, teachers and students talk to each other: what do you make of this? Are you thinking what I’m thinking? The models of intersubjective engagement and institutional management presented earlier raise questions about human relations within an organization, and ways in which curriculum change, dialogue, and accommodation of technology might be approached. The space for dialogue can be removed however, if management (or government) holds to a particular vision of the future, together with a means of implementing that future transforming what they believe to be the ‘present’. Such an approach risks ignoring the space within which many different versions of possible ‘futures’ and possible ‘presents’ exist in the many heads of teachers and learners. Rather than embracing the uncertainty and dialogic potential of asking, ‘Whose present? Whose future?’, the imposition of a monocultural future risks alienation.

With its focus on realizing envisaged futures, implementation research has become influential as a framework for establishing institutional curriculum change around digitalization and a move from present to future (Fitz et al. 1994; Century and Cassata 2016). Emerging alongside experiences of implementing improvements in industry and healthcare, implementation research is loosely influenced by design-based research albeit with specific implemented outcomes as objectives. Since even in ‘simple’ implementation situations (like implementing a vaccine program, or a new computer system), intersubjective dynamics and organizational dynamics give rise to complex issues of trust and risk (Rose and Schlichter 2013; Jones 2008), in education, where intended outcomes are contested, linearity in the implementation research ignores the uncertainty of a multiplicity of possible futures and presents.

In contrast to a linear model of implementation, the Global Scientific Dialogue course began with us asking *whose* ‘present’ and *whose* ‘future’. This meant acknowledging that in all cases, both present and future are constructed by observers who are stakeholders in the education system: what are perceived as present deficiencies are conceptually tied up with imagined futures, and different stakeholders will construct both present and future in different ways based on their own experiences, biographies, and skills. This matters when the key stakeholders who deliver new initiatives are not those who designed them, and where those who decide on the necessity for this envisage a linear form of implementation. This means that inevitably there is uncertainty and ambiguity in the construction process, and that this must be resolved in the communication dynamics between stakeholders. In our case, creating the context for dialogue about the challenges of the course, technology, and metacurriculum meant that a co-designed approach among teachers was essential in order to create the conditions for dialogue about rationale and ambition.

Having said this, to get the ball rolling with any new initiative requires some kind of initial assertion. We began with a ‘test course’—a 4-day long ‘initiation’ where a community of 20 teachers began to work together on a cut-down prototype of the course. Having participated in this, teachers would meet regularly thereafter to prepare the ‘full’ course for the students. The teachers were taken from a range of disciplines (soil science, economics, management, tourism, biology) and most of them had not worked together before. This group worked together to deliver the course to students, with many teachers continuing as part of the group over the period of 4 years. The student cohort size varied from 120 to 200 learners, with the course running in a concentrated block of 8 days over 2 weeks.

A sample of the teachers (*n* = 8) were interviewed in depth about their experiences over the 4 years of the course in 2021. Asked how they felt at the beginning of this process, they said that there was some confusion as to how the course connected with other disciplines, subject areas, and activities: ‘it was hard switch the students to a different rhythm [in their education]’, one teacher commented. Another noted that it was the teachers who had more learning to do: ‘the teachers are moving to a future which is already present for the students’. One of the common requests during the design phases of the course was for more content—i.e., that teachers were uncomfortable coordinating activities where the learning resources existed to provoke dialogue about wicked problems, creativity, and technology, rather than being ‘taught’. The dialogue did begin among the teachers though—and despite the initial confusion, these teachers stayed with the course expressing the view (in interview) that as they got more familiar with it (and had a greater say in what was done) it was much easier to see how the elements came together. It was considerably different from anything else they did at the university.

In describing their experiences over this period, ‘listening’ was a dominant theme in the teacher reflections. Representative comments include: ‘We started listening to the students’, ‘I started listening more, and constructed a dialogue in a different way’, ‘I was interested in listening to alternative points of view from my students’, ‘Students talked more and I listened and sometimes directed their dialogue’, ‘I listen more and talk less so I better understand students needs’.

The technological emphasis of the course from the first 2 years paved the way for a redesign of the course under Covid-19. The pandemic was instrumental in bringing people together to address the restrictions of coordinating dialogue online, while the technological themes proved to be a catalyst for innovation which retained the original spirit of the course. Reflecting on this transition, one teacher said: ‘This time we entered into the ecology of the course very easily. I realized how I could use the course in my future work with my students. I started to refer to the course in my work’.

Monthly design meetings were used to test various ideas with the teachers. A further benefit was that where the use of technology in the first two iterations involved dealing with audio/video equipment which sometimes did not work, the shift to online-only meant that many of these issues became irrelevant as video was the principal means of communication, while coordination of a large cohort of learners proved easier in Microsoft Teams than it had been in the physical environment.

In interviews, this 3^rd^ year of the course in 2020 allayed any remaining doubts about the coherence of the different activities. The online format, with its emphasis on online team-working, media creation, and exploration of technical skills, created the conditions where it (as one teacher put it) ‘let the teachers learn more about themselves and the students’. The students were assessed through online presentations for which they were given a high degree of flexibility to express themselves. Students presented videos reflecting what they found interesting: these contained features including AI-generated content, examples of data analysis, animation, music, poetry, and even an interactive presentation as a Python program.

Covid-19 produced a lot of cooperation. One of the consequences of this was a reflection on the teacher’s role. There was ‘more reflection in the classroom’ and discussion about teaching among teachers, with one teacher commenting that ‘the course allowed me to reaffirm my vision of a teacher, not as a director, but as facilitator’. There was no longer a question of ‘whose future?’ but rather a coordination of understanding between the teachers about both the ‘present’ and the ‘future’.

The approach to team teaching, dialogical pedagogy, and flexibility of assessment all contributed to this. This showed itself through further co-design sessions where teachers were open to exploring new possibilities on the understanding that the educational objective was to get students to talk to each other. The course design using Microsoft Teams used a wide variety of technologies, deploying a kind of ‘tool-oriented’ pedagogy. This facilitated deeper engagement with the students and (some teachers felt) an increased self-awareness. The online-only experience provided a new dimension in understanding the relationship to technology, and teachers spoke of how some activities appeared to be deeper in the online medium (particularly programming, and in the extensive use of video in learning activities) where, while the rich dynamics of the classroom could not be reproduced in the same way, other forms of collaboration took their place including collaborative video-making, alongside opportunities for more rapid formative feedback to students.

## Measurement and Metacurriculum

Collegiality has been a central ingredient in the implementation of our metacurriculum. Referring back to Fig. 3, this is an aspect of the coordination of uncertainty among teachers which facilitated a move beyond the security of disciplines, towards a more transdisciplinary way of operating (Fig. 4). This raises the question of what might be said about the learners, and what data might support a similar movement in their engagement with the course activities.

If a metacurriculum is a dynamic process existing as a relation between discipline-specific engagement and transdisciplinary engagement (Fig. 4), we need to study the relations learners have with different topics, how those topics generate responses from learners, and how different responses to different topics relate to one another. While classroom discussion is difficult to examine in detail, we devised an activity and technique which would:

- Create a lot of data about student reflection in relation to a range of topics; and
- Provide a way of integrating the different themes and experiences of the course.

Our approach was based on the technique of comparative judgement (CJ) and was used as a design feature from the beginning of the course. CJ is normally used as an assessment approach (Pollitt 2012), but here we used it as a way of collecting rich data about student preferences and reflections, partly influenced by the work of Milner (2021) who developed a ranking-based method of educational inquiry in the 1930s. Using CJ, rankings can be established across a range of documents (in our case, 93 short pre-prepared texts) through a process of pair-wise comparison. For each pair, chosen freely by the students, they were asked to write about which of the pair they found more interesting and why. This produces both a ranking of topics, and an indication of the level of ‘fit’ between the students’ reflections and the topic documents, using a cosine similarity measure.

This exercise was repeated for each year of the course. The data presented here relates to the iteration of the course from 2021, when 120 students participated in the activity during the course and each student had been asked to make at least 2 comparisons. Students represented the different Master’s courses of the school, including economics, management, and tourism. The emphasis of the data collection was in producing a collective ranking of documents which were anonymized (this was partly because student data was later used as the basis for collective data analysis exercise to produce word clouds as part of the course), so there was no need to track individual activity with the software. Not everyone contributed data, but the exercise elicited 223 records, mostly with surprisingly extensive reflections (the average length of response was 530 characters).

The pre-prepared texts were organized around subjects ranging from AI and blockchain to psychotherapy and quantum mechanics. The exercise of comparison and reflection served the dual pedagogic purpose of encouraging the students to read around the subjects they were engaging in classroom, while providing some cohesion to the range of topics and activities which they encountered. Data collected from this exercise provides an indicator of both the level of intellectual engagement that the students had with the topics and the emerging structure of their understanding (Johnson et al. 2020)—particularly as it relates to the journey from the specifics of their disciplines to transdisciplinary issues. Further indicators of student experience were provided by a course evaluation questionnaire completed by 50% of the students.

The texts presented to students were organized into eight categories relating to the course. Within each heading, short statements and questions were designed to prompt student reflection. Each topic had associated ‘key questions’. For example: ‘Could you live without the Internet or your phone?’, ‘How can a brain describe a brain?’, or ‘Do technological advances create more things to worry about?’. Wicked problems were addressed in articles on global warming (‘Are ecological problems the result of bad decisions?’), bullying or inequality, while other articles related to fields in which the students were studying, including business (‘What has technology done to business?’), management (‘What is the difference between a beehive and a business?’), and tourism (‘What is leisure?’). Students were free to choose any pair of documents from any of these categories to compare. While the documents mapped a space between specific disciplinary areas (particularly those concerning technology) and broader issues from sociology, psychology, ecology, and phenomenology, comparison highlighted the uncertainties of the difference between specific and transdisciplinary topics.

The comparison exercise elicited thoughtful responses from students, producing data not only in the text of their responses, but in the specific documents chosen (and those not chosen), the broader disciplinary areas of those topics, and in the overall ranking of the popularity of particular topics over others. Some comparisons were made where two documents from the same category were chosen (*n* = 102), while other comparisons were made between topics (*n* = 120). Considering that some topics were more transdisciplinary than others, this pattern of comparison can be mapped onto the range between subject-specific content and transdisciplinary content. The comparison data is a measure of the student’s selection processes: what grabs their attention? How do they make distinctions between different kinds of content and different questions?

Given this, simply counting the topic categories which were most popular is informative in terms of where the weight of interest sits with regard to the metacurriculum journey. Table 1 shows the ranking of topics across all selections.

**Table 1:** Number of comparisons by category (whole dataset)

Category	No. of comparisons
Technology	55
Creativity	36
Intersubjectivity	34
Interdisciplinarity	25
Science	24
Business	23
Data	14
Education and Science	9

It is noteworthy that those subjects which are most interdisciplinary (technology, creativity) gained the most interest and attention from the students, while those which were most specific to disciplines (business and data) gained the least. This list may be broken down into:

- Those comparisons which were made across different topic areas—thus maximizing the potential difference between the compared documents (Table 2);
- Those comparisons which were made within the same topic areas—where there was potentially a lot of similarity between the compared documents (Table 3).

**Table 2:** Number of comparisons by category where documents of different categories are compared

Category	No. of comparisons
Intersubjectivity	25
Creativity	20
Interdisciplinarity	16
Science	16
Technology	15
Business	11
Data	9
Education and Science	6

**Table 3:** Number of comparisons by category where documents of the same category are compared

Category	No. of comparisons
Technology	40
Creativity	16
Business	12
Intersubjectivity	9
Interdisciplinarity	9
Science	8
Data	5
Education and Science	3

With regard to (a), it is clearer that transdisciplinary subjects were more favored when compared to documents of a different type (Table 2).

Meanwhile, possibly owing to the disproportionately large number of comparisons made about technology, the popular comparisons made within the same category produced a different ranking (Table 3).

The focus on technology reflected the principal activities in the taught sessions. Students had engaged in sessions on AI, video and multimedia production, social media, interdisciplinarity, programming (Python and Javascript), and communication. In the evaluation survey of the 2021 cohort, of 60 responses, students valued the technical input most highly (video production, 59%; AI, 69%; social media, 44%). However, despite the focus on technology within the taught sessions, and the students’ enjoyment of this, the fact that the cross-topic comparison favored intersubjectivity, creativity, and interdisciplinarity suggests that these broader transdisciplinary themes, when pitted against more specific technological themes, were more interesting to the students.

In addressing these broader themes in the course, questions, problems, objects, and sometimes people in the form of external experts were presented in the environment alongside videos, tools, and activities, each of which articulate uncertainties and contradictions. These interventions were intended to amplify ‘uncertainty 2,’ which in the first 2 years of the course took place in the classroom, and in the next 2 successive years took place online. In introducing teachers and students to new technologies (for example, machine learning), the uncertainty inherent in the different understandings of the technology could be amplified by engaging both students and teachers in activities with Google’s ‘Teachable Machine’,^2^ Python-based analysis of data, or exploring and explaining the behavior of the Oxford image recognition models^3^ and then engaging them in discussion about surveillance, manipulation, distraction, and fake news.

## Measuring the ‘Fit’ in Student Reflection with Cosine Similarity

What happens to a student (or a teacher) when faced with some aspect of environmental uncertainty which they have to resolve by justifying which of two documents is preferred? In terms of the model of Global Scientific Dialogue (Fig. 3), the presentation of the documents relates to ‘uncertainty 2’ (external uncertainty), while the resolution must result in communication arising from a need to resolve ‘uncertainty 1.’ When asked to make a choice between two documents and to justify ‘why $a$ rather than $b$,’ it is perhaps to be expected that the student’s justification might have more similarity to the text that they preferred than to the one they rejected. This similarity can be measured with statistical techniques, and these (mostly) correlated with expectations. However, the statistical similarities between the documents chosen and the justification raise deeper questions about meaning and expectation in the students which we discuss below.

Cosine similarity provides one way of quantifying the degree of similarity between two texts. It computes its value from two or more texts (for example, ‘Digitalization is the future of education’ and ‘I think the statement about digitalization is interesting’) by first analyzing individual terms (words) as a grid that indicates which document contains which words. When plotted on a graph of each term against its frequency in each document, an angle is formed between different words in the documents. The smaller the angle, the more similar are the documents, and the cosine similarity is closer to 1. In the case of the above example, the cosine similarity is 0.202.

In our analysis, cosine similarity scores have been assigned to each of 224 comparisons and each text of the original documents that were compared. Table 4 shows the mean values of these cosine similarity scores across each category. The data suggests that the justification text is always closer to the compared document than the cosine similarity with the documents that were rejected (so the difference column is positive, albeit in some cases small). However, in comparison to Table 1, the ordering by cosine similarity is markedly different from the ranking of popularity of topics, although with the exception of ‘Education and Science’, interdisciplinary subjects still dominate.

**Table 4:** Cosine similarity across all documents ranked by similarity according to document category

Subject	Cos-sim (selected)	Cos-sim (unselected)	Difference sel.-unsel	Weight	Variance
Education and Science	0.214	0.071	0.143	9	0.020
Intersubjectivity	0.180	0.118	0.062	34	0.018
Creativity	0.176	0.084	0.092	36	0.014
Interdisciplinarity	0.148	0.068	0.079	25	0.012
Science	0.145	0.073	0.072	24	0.008
Technology	0.131	0.066	0.066	55	0.004
Business	0.131	0.095	0.035	23	0.011
Data	0.127	0.065	0.062	14	0.006

The high cosine similarity for Education and Science is based on a small sample, and the contributions of one particular student who chose to defend their document choice by summarising the contents of both documents before defending their judgement:

Brief retelling: 1 Article 1: Technology. About how technology has changed our world and how it will change further, in particular the labor market.

2 Article 63: Educational Assessment. The author discusses the education system and concludes that grades are a way to maintain trust in the university. Thanks to the issuance of marks, it is possible to select students and issue diplomas only to those who are worthy. It is possible to meet the expectations of a teacher without factual knowledge and, accordingly, get good grades, but this rarely happens.

This retelling produces a high similarity score, and is clear evidence of some thoughtful reflection on what they were asked to compare. After this retelling, the student argued for the selection of the document about education, saying:

In general, in my ‘ideal education system’ there would be credits so that employers understand that students graduated from university and they really tried to acquire knowledge, but there would be no grades, because for me personally it is a lot of stress and a ‘red diploma’ [university certificate] often does not speak about the level of education of the student.

The extent to which the cosine similarity score reflects the level of depth of reflection raises a deeper question: is the preferred document chosen because its pattern of communication already maps onto the pattern of communication within the student prior to them writing their justification? Is the mechanism that selects utterances within the students (in managing uncertainty 1) better suited to processing and commenting on the information in the preferred document?

In communication theory (Luhmann 1996), utterances have to be selected. For two people making similar utterances (here, an author and a learner), there is a possibility that their selection mechanisms operate in a similar way. To understand someone’s utterance as meaningful is then to recognize that they select utterances in a similar way. In this sense, an agreement is what Schutz calls a ‘tuning-in to the inner world of the other’ (Schutz 1967). In suggesting (with Leydesdorff et al. 2017) that techniques like cosine similarity (or Shannon mutual redundancy (1949), in Leydesdorff’s case) might provide an indicator of this mutual tuning-in is to suggest that there are ways in which the construction of meaning can be quantified. Within the course systems diagram in Fig. 3, this mutual tuning-in, or coordination of expectations, can be understood as the function of the relationship between the ‘anticipatory mechanism’ and the ‘internal management’ of each actor.

Further break-down of the cosine similarity tables along the same lines as Tables 2 and 3 shows that the popularity of topics indicated in Table 2 is reflected in cosine similarities of the responses to those topics (apart from the anomaly described above). The interdisciplinary topics have the highest cosine similarity scores. This pattern is consistent irrespective of whether the documents were chosen within the same topic (Table 5) or across different topics (Table 6). This suggests that once the student has made their selection, their focus is on defending their choice of document over the other document, and writing text which reflects their favored document.

**Table 5:** Cosine similarity within-topic comparisons

Subject	Cos-sim (selected)	Cos-sim (unselected)	Difference sel.-unsel	Weight	Variance
Intersubjectivity	0.276	0.133	0.142	9	0.029
Education and Science	0.184	0.093	0.092	3	0.038
Creativity	0.178	0.101	0.077	16	0.006
Business	0.160	0.069	0.091	12	0.014
Data	0.156	0.089	0.066	5	0.005
Science	0.147	0.071	0.076	8	0.008
interdisciplinarity	0.126	0.074	0.053	9	0.012
Technology	0.123	0.069	0.054	40	0.004

**Table 6:** Cosine similarity outside-topic comparisons

Subject	Cos-sim (selected)	Cos-sim (unselected)	Difference sel.-unsel	Weight	Variance
Education and Science	0.229	0.066	0.163	6	0.010
Creativity	0.174	0.072	0.101	20	0.020
Interdisciplinarity	0.159	0.063	0.096	16	0.011
Technology	0.154	0.060	0.094	15	0.005
Intersubjectivity	0.146	0.110	0.036	25	0.010
Science	0.144	0.074	0.070	16	0.008
Data	0.111	0.045	0.067	9	0.006
Business	0.099	0.130	-0.031	11	0.007

If the cosine similarity score between student reflections and the original texts is an indicator of similarity between selection mechanisms for utterances (i.e., between an author and the student), then the weighting of the number of students with similar choices is a further indicator of the likelihood that within a body of students, certain conversations about certain documents will stimulate an ongoing dialogue. While the present analysis did not track individual student choices (partly for reasons of data protection), had we done so then it would be possible to measure both the range and the overlap of interest across the cohort. This would give an analytical focus to the metacurriculum as a dynamic space within which connections between topics and people grow organically.

## Conclusion: Education, Meaning, and Digitalization

Seeing digitalization in education as the linear implementation of an imagined future from an imagined present is an illusion. Asking about whose constructions matter, and how diverse constructions of ‘present’ and ‘future’ can be coordinated, can help institutions align themselves better to a fast-changing technological environment, taking learners and teachers on a journey that embraces new practices. Our experiences with a large group of teachers over a period of 4 years have shown this can be done with a combination of team teaching, co-design, dialogical education, active learning, tool-based pedagogy, and flexibility in curriculum and assessment. We have argued that a metacurriculum, as a means of navigating the space between disciplinary specifics and technological transdisciplinarity, entails such a process of coordinating expectations as constructions between and among teachers and learners.

The Global Scientific Dialogue approach, as an example of a metacurriculum, looks to create the conditions where possible constructions—both of the present and the future—can be explored by foregrounding the essential uncertainties that are presented by technology. In this model, technology is not a thing to be taught, but a means by which uncertainties can be generated and explored, thus prompting deeper dialogue. This approach appears to have been successful in encouraging student reflection and practical exploration of technology, while engaging a large group of teachers in supporting the program and facilitating these discussions. While the shocking events of 2022 have ended the present collaboration, the course continues and we hope that some more generic lessons might be applicable elsewhere.

We have made the case that dialogue is the appropriate educational response to the increasing complexity of the technological environment. In its focus on dialogue, Global Scientific Dialogue highlights the importance of facilitating the student and teacher journeys from the specifics of their disciplinary studies, towards the transdisciplinary opportunities presented by technology. This is not to ignore disciplinary specialization—philology and quantum mechanics belong in disciplines—but it is to see a holistic dynamic which connects specialization and the traditional structures of the university with the elastic movement of inquiry in an increasingly technological world.

Our initial data from the comparison activity provides some rudimentary indication of the intellectual, dialogical, and structural nature of this elastic inquiry. It suggests that there is both a need, and the means to find ways in which personal interests of students can be deepened through dialogical and technological engagement—which has always been the principal objective of a metacurriculum.

## Footnotes

1 See https://www.dvfu.ru/schools/school_of_economics_and_management/global-scientific-communications/ (in Russian). Accessed 1 July 2022. 

2 See https://teachablemachine.withgoogle.com/—a ‘no-code’ approach to creating machine learning models designed for children. Accessed 1 July 2022. 

3 See https://www.robots.ox.ac.uk/~vgg/—the Visual Geometry Group (VGG) model is a commonly-used machine learning architecture for image recognition. Accessed 1 July 2022. 
